# Core-shell nanocarriers with high paclitaxel loading for passive and active targeting

**DOI:** 10.1038/srep27559

**Published:** 2016-06-09

**Authors:** Zhu Jin, Yaqi Lv, Hui Cao, Jing Yao, Jianping Zhou, Wei He, Lifang Yin

**Affiliations:** 1 Department of Pharmaceutics, School of Pharmacy, China Pharmaceutical University, Nanjing, 210009, P.R. China

## Abstract

Rapid blood clearance and premature burst release are inherent drawbacks of conventional nanoparticles, resulting in poor tumor selectivity. iRGD peptide is widely recognized as an efficient cell membrane penetration peptide homing to α_V_β_3_ integrins. Herein, core-shell nanocapsules (NCs) and iRGD-modified NCs (iRGD-NCs) with high drug payload for paclitaxel (PTX) were prepared to enhance the antitumor activities of chemotherapy agents with poor water solubility. Improved *in vitro* and *in vivo* tumor targeting and penetration were observed with NCs and iRGD-NCs; the latter exhibited better antitumor activity because iRGD enhanced the accumulation and penetration of NCs in tumors. The NCs were cytocompatible, histocompatible, and non-toxic to other healthy tissues. The endocytosis of NCs was mediated by lipid rafts in an energy-dependent manner, leading to better cytotoxicity of PTX against cancer cells. In contrast with commercial product, PTX-loaded NCs (PTX-NCs) increased area under concentration-time curve (AUC) by about 4-fold, prolonged mean resident time (MRT) by more than 8-fold and reduced the elimination rate constant by greater than 68-fold. In conclusion, the present nanocarriers with high drug-loading capacity represent an efficient tumor-targeting drug delivery system with promising potential for cancer therapy.

PTX is an efficient chemotherapeutic agent for a broad range of solid tumors, including ovarian cancer, non-small cell lung cancer and breast cancer^1^,^2^,^3^. However, its clinical implementation is limited due to poor solubility in aqueous solutions; non-specific distribution throughout the body that causes insufficient penetration into tumors; toxicity to healthy tissues, which limits the dose and frequency of the treatment; and cancer cell resistance^4^,^5^,^6^,^7^.

NCs, which have a 1–1000 nm dimensional range, are colloidal drug carrier systems consisting of a lipid core and a thin polymer membrane^8^,^9^. NCs are considered to be a type of “reservoir” system^10^,^11^. Compared with other nanosystems^12^,^13^,^14^,^15^, the NCs not only encapsulate both hydrophilic and hydrophobic drugs with high drug-loading capacity but also display great potential for enhancing the antitumor effects of drugs while having a low toxicity^16^,^17^,^18^,^19^,^20^,^21^. Moreover, the properties of NCs, such as size, charge and surface functionality, can be tuned easily by modifying their surface chemistry^22^,^23^,^24^.

In a previous report^8^, we developed a novel and simple method to prepare NCs based on nanoemulsion-templates stabilized by β-lactoglobulin (β-LG), in which the nanoemulsion-template generation and shell crosslinking were simultaneous. No surfactants or organic solvents were used in the preparation of the NCs, indicating excellent biocompatibility. More importantly, the NCs with core-shell structures had extremely high stability and drug-loading capacity for lipophilic drugs. Furthermore, the presence of carboxyl and amine moieties on the surface makes it easy to modify the NCs with various ligands for targeted drug delivery, bioimaging and therapeutics.

α_v_β_3_ integrin is a cell-adhesive receptor that is overexpressed on tumor vessels but not on normal tissue vessels^25^,^26^,^27^, whereas Neuropilin-1 (Nrp-1) is a transmembrane receptor that is highly expressed in a variety of human carcinoma cells and is correlated with angiogenesis and vascular permeability^28^. iRGD (CRGDRCPDC), a cell-penetrating peptide, first binds to the α_v_β_3_ integrin, exposing a binding motif for Nrp-1 through proteolytic cleavage and mediating receptor-related endocytosis^28^,^29^. Thus, the iRGD-modified nanocarriers would have enhanced tumor-targeting activity due to the iRGD-integrin interaction.

Hence, we hypothesized that such NCs not only could well encapsulate PTX with high drug-loading but also could be easily modified with iRGD, therefore improving PTX delivery and achieving active targeting for tumor therapy. To obtain a proof of concept, various studies were conducted to characterize the nanocapsules, evaluate the *in vitro* cytotoxicity, study the internalization mechanism, determine the pharmacokinetic and biodistribution profiles, and, finally, evaluate the *in vivo* antitumor activity. Interestingly, significant improvement in pharmacokinetics of PTX was obtained by NCs, exhibited as markedly prolonged circulation-time in blood and increased AUC. As expected, the NCs exhibited better antitumor activity because of the high drug-loading capacity, prolonged systemic circulation, and enhanced accumulation and penetration into tumors, which was further improved by iRGD modification. The present nanosystem, due to its high drug-loading efficiency and easy modification with ligands, is a promising platform for enhancing delivery of PTX.

## Results

### Preparation and characterization of NCs and iRGD-NCs

**Figure 1:**
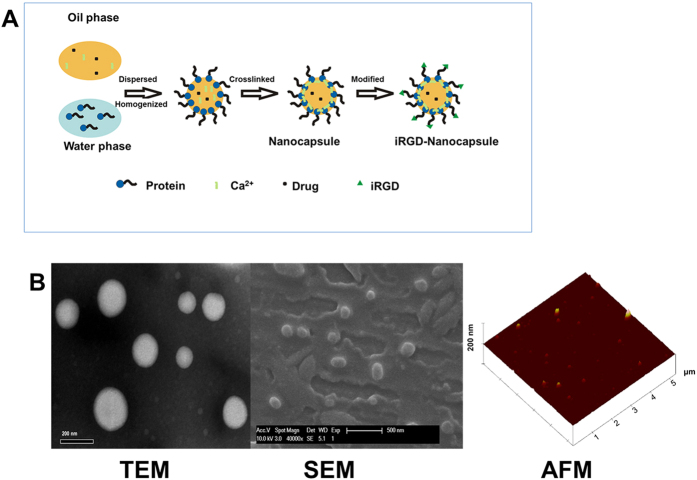
(**A**) Scheme of the preparation process of NCs and iRGD-NCs. **(B**) TEM, SEM, and AFM images of iRGD-NCs.

The preparation procedure of NCs and iRGD-NCs is shown in Fig. 1A. The mean particle size of the NCs and iRGD-NCs was 180 and 195 nm with polydispersity index (PDI) values of 0.086 and 0.138 (see Supplementary Table S1 and Supplementary Fig. S1), respectively. Transmission electron microscope (TEM) observation indicated that the iRGD-NCs were uniform spheroids with diameters ranging from 150 to 220 nm (Fig. 1B), in general in line with the results from the dynamic light scattering (DLS) measurement. It was noted that there were some very small nanoparticles shown in TEM image due to the destruction of nanoparticles in sample preparation or aggregation of protein in aqueous conditions in preparation of NCs. The scanning electron microscope (SEM) and atomic force microscope (AFM) images further confirmed that the iRGD-NCs present as spherical morphology in uniform particle sizes with high dispersity and there was no aggregation or adhesion among the iRGD-NCs (Fig. 1B).

Theoretically, the NCs were conjugated with iRGD using maleimide polyethylene glycol-2000 succinate ester (MAL–PEG2000–NHS) as a bridge because the –MAL groups could react with the –SH groups of iRGD at pH 7.1 and the –NHS groups could react with the –NH_2_ groups of β-LG at pH 8.5^30^. To confirm the connection between iRGD and the NCs, we performed the spectroscopic analysis of ^1^H-NMR, fourier transform infrared (IR) and ultraviolet-visible (UV-Vis). As shown in the ^1^H-NMR spectrum, a characteristic peak of the –MAL group in PEG was found at around 7.0 ppm (see Supplementary Fig. S2A), while the characteristic peak of –MAL disappeared in the spectrum of iRGD-PEG2000-NHS (see Supplementary Fig. S2B), suggesting that the –MAL groups had reacted with the SH group of iRGD^31^. The amplification of υ_NH_ (3500 nm) and υ_C=O_ (1700 nm) in the IR spectrum were observed (see Supplementary Fig. S3A), confirming a connection between iRGD and MAL–PEG2000–NHS^32^. A colorimetric method using UV-Vis was performed to identify the connection between iRGD-PEG2000-NHS and the NCs. The PEG linker could react with ammonium ferrothiocyanate and was subsequently moved from the aqueous phase to the trichloromethane phase, resulting in an absorption peak at 500 nm in the UV-Vis spectrogram^33^. As depicted in Fig. S3B,C, the characteristic peak of PEG at around 500 is highly visible in the iRGD-PEG2000-NHS modified NCs, and more profound colour reaction is observed from the modified NCs. Therefore, the connection between iRGD and NCs was further confirmed.

The drug loading capacity (DL, %) and encapsulation efficiency (EE, %) were 12.91 ± 0.19% and 86.04 ± 1.29%, respectively, for the NCs and 12.48 ± 0.37% and 83.18 ± 2.44%, respectively, for the iRGD-NCs. Generally, the DL in traditional nanoparticles is not greater than 10%^19^. Herein, the NCs had high PTX loading, exhibiting its great potential application in anticancer therapy.

The *in vitro* drug release profiles of the PTX formulations are present in Fig. S4. About 60 percent of PTX was released from Taxol^®^ (control) 24 h later while the drug release from iRGD-NCs or NCs was around 30 percent, without initial burst release. It thus indicated that little of the drug was adsorbed on the nanoparticle surface and the drug was well encapsulated in the lipid-cores, which were responsible for the sustained drug release over time. Moreover, the drug release from iRGD-NCs and NCs was similar, suggesting that the conjugation of iRGD do not affect the drug release. In general, the drug release from the present nanocarriers was controlled by the diffusion through the core and subsequently across the shell, while the latter was a rate-limiting step for the release.

### Cellular uptake, internalization mechanism and intracellular tracking

**Figure 2:**
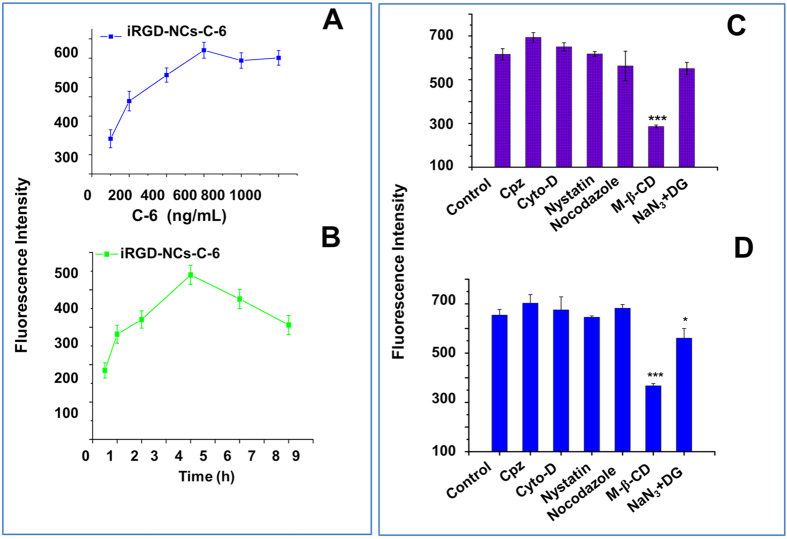
Cellular uptake and internalization mechanism. **(A**,**B)** Intracellular fluorescence intensity of C-6-loaded iRGD-NCs incubated with cells at various concentrations and times. Fluorescence intensity of A549 cells incubated with 400 ng/mL C-6-loaded **(C)** NCs or **(D)** iRGD-NCs for 2 h in the presence of various inhibitors, respectively. *p < 0.05, ***p < 0.001 *vs* control (n = 3).

Cellular uptake was examined in A549 cells to assess the cell membrane penetration activity of iRGD and the internalization mechanism. Compared with coumarin-6-loaded NCs (NCs-C-6), a higher intracellular accumulation of iRGD modified NCs-C-6 (iRGD-NCs-C-6) was observed at the C-6 concentration of 400 ng/mL after 2 h of incubation (see Supplementary Fig. S5). A quantitative analysis performed with flow cytometer (FCM) revealed that the cellular uptake of iRGD-NCs-C-6 was concentration-dependent until the concentration reached 600 ng/mL; after that, the uptake plateaued (Fig. 2A). As time elapsed, the cellular uptake of iRGD-NCs-C-6 increased in the first 4 h-period and decreased in the second 4 h-period (Fig. 2B). The results were in line with the characteristics of endocytosis, which occurs in a concentration- and time-dependent manner^34^,^35^.

To clarify the endocytic mechanism of NCs-C-6 and iRGD-NCs-C-6, inhibitors were used to block the internalization pathways. The inhibitors included chlorpromazine (Cpz), cytochalasin-D (Cyto-D), nystatin, nocodazole, methyl-β-cyclodextrin (M-β-CD) and NaN_3_ with deoxyglucose (NaN_3_ + DG), which block clathrin-mediated endocytosis, macropinocytosis, thecaveolin-internalization process, microtubule-related internalization, and cholesterol-dependent and energy-dependent mechanisms, respectively. Of the inhibitors, the cellular uptake of NCs-C-6 was inhibited significantly only by M-β-CD (Fig. 2C), while NaN_3 _+ DG and M-β-CD produced significantly suppressive effects on internalization of iRGD-NCs-C-6 (Fig. 2D), indicating that the internalization pathway was affected by the ligand.

**Figure 3:**
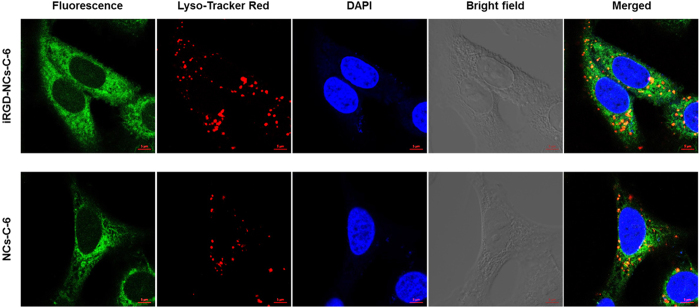
Intracellular distribution and localization of iRGD-NCs-C-6 and NCs-C-6 in A549 cells. The cells were incubated with 400 ng/mL C-6-loaded iRGD-NCs or NCs for 2 h. The nuclei (blue area) and lysosomes (red area) were stained with DAPI and Lyso-Tracker Red, respectively. The scale bar is 5 μm.

The intracellular distribution and localization of iRGD-NCs-C-6 and NCs-C-6 in A549 cells were detected using confocal laser scanning microscopy (CLSM) (Fig. 3). The nanocarriers (green) were distributed around the nucleus (blue) and spread widely inside the cell. The enhanced fluorescence intensity of the cells treated with iRGD-NCs was observed because iRGD facilitated the cell penetration process. To track intracellular delivery, the lysosomes were labeled with Lyso-TrackerRed (Fig. 3). When the green fluorescence and red fluorescence (lysosomes) overlap, a yellow area is observed in the merged image, illustrating both part of iRGD-NCs-C-6 and NCs-C-6 located in the lysosomes. Accordingly, the internalization occurred through an endolysosomal pathway mediated by endocytosis.

### *In vitro* cytotoxicity and cell apoptosis

To evaluate the cytotoxicity of the nanocarriers, the cell lines including A549, MDA-MB-435, SGC-7901 and H22 cells were incubated with blank NCs (see Supplementary Fig. S6A) or iRGD-NCs (see Supplementary Fig. S6B) at 37 °C for 24 h. Irrespective of the concentration of nanocarriers, the viability of MDA-MB-435, SGC-7901 and H22 cells was greater than 90%. A549 cells were more sensitive to the blank nanocarriers, especially to the high concentration; however, the viability for all tested concentrations was not less than 80%. It therefore indicated that the nanocarriers did not exert any significant cytotoxicity to the cells^36^.

The cell viability after incubation with PTX-loaded iRGD-NCs (iRGD-NCs-PTX), PTX-loaded NCs (NCs-PTX) and Taxol^®^ for 48 h, where the anti-proliferation activity occurred in a dose-dependent manner, is shown in Fig. S6C. The IC_50_ values for iRGD-NCs-PTX, NCs-PTX and Taxol^®^ were 0.425, 0.652 and 0.794 μg/mL, respectively. These data suggested that NCs enhanced the cytotoxicity of PTX against cancer cells, while the iRGD-NCs appeared to be more potent, as iRGD improved the internalization of the NCs.

An Annexin V-FITC/PI kit was used to stain the early and late apoptotic cells, and the percentage of apoptotic cells was measured using FCM (see Supplementary Fig. S6D). The total percentage of early and late apoptosis in H22 cells was 13.43 ± 1.46%, 9.15 ± 2.01% and 2.68 ± 0.39% for iRGD-NCs-PTX, NCs-PTX and Taxol^®^, respectively, thereby indicating that iRGD-NCs-PTX was most efficient in inducing apoptosis in cancer cells.

### Tumor spheroid penetration

We have confirmed the excellent anti-tumor activity of the present nanocarriers on human tumor cell lines *in vitro*; however, the performance of a 2-dimensional model could not simulate real solid tumor tissue. Ideally, the nanocarriers should not only penetrate across the blood vessels but also penetrate deeply into the inner part of tumor to deliver drugs. Herein, the 7-day tumor spheroids were used to incubate with C-6 labeled iRGD-NCs or NCs at 37 °C, and the fluorescence images of the spheroids observed by CLSM were taken 4 h later. Figure S6E shows the images of tumor spheroids after being incubated with iRGD-NCs-C-6 or NCs-C-6 for 4 h. The two nanocarriers penetrated into the inner part of the tumor spheroids, but stronger fluorescence intensity was observed with the iRGD-NCs-C-6, thereby indicating that iRGD facilitated the penetration of the NCs into the tumor spheroids.

### Pharmacokinetics in rats

As depicted in Fig. S7A, Taxol^®^, iRGD-NCs-PTX, PTX-loaded PEG2000 modified NCs (PEG-NCs-PTX) and NCs-PTX had a steep slope in the plasma concentration versus time curves, and the concentration of PTX decreased rapidly to a low level within 2 h, suggesting that the nanocarriers were easily distributed into other tissues from the blood. As shown in Table 1, the values of the AUC of NCs-PTX and iRGD-NCs-PTX were approximately 4.3 and 5.8-fold greater than that of Taxol^®^ (p < 0.05), demonstrating enhanced bioavailability. It was noting that the AUC of NCs-PTX was also significantly more than that of PEG-NCs-PTX, confirming the enhanced prolonged circulation from the former. Consequently, it was expected that the improved long circulation time could lead to an increased accumulation of the nanocarriers in tumor tissues via passive and active pathways, thereby enhancing the antitumor activity.

### *In vivo* biodistribution and tumor targeting

**Figure 4:**
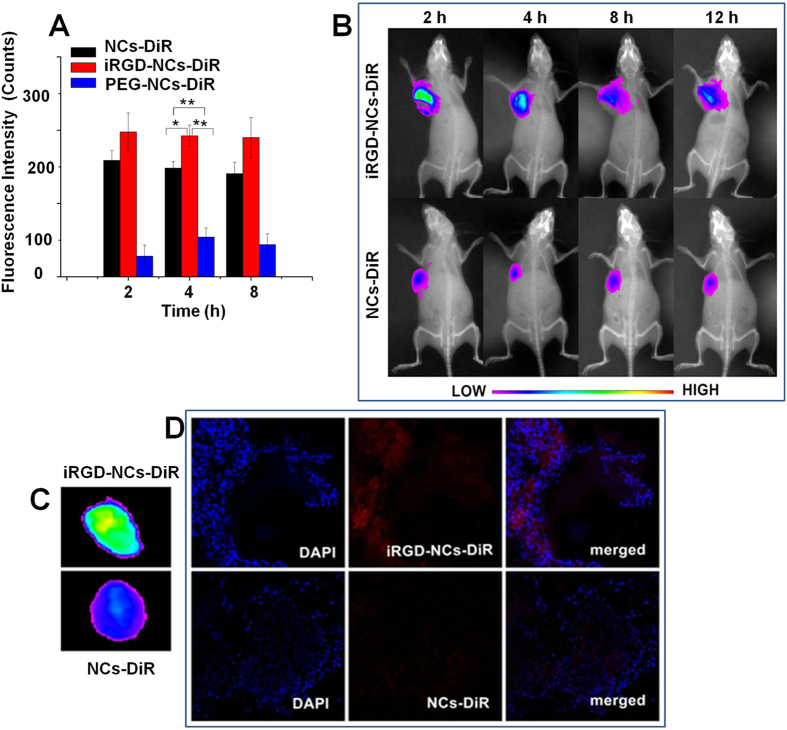
*In vivo* biodistribution and tumor targeting. **(A)** *In vivo* distribution of DiR-loaded iRGD-NCs, PEG-NCs and NCs in tumor tissue at 2, 4 and 8 h after administration. The fluorescence intensity represents the quantity of iRGD-NCs, PEG-NCs and NCs in the tissues (n = 5). *p < 0.05 and **p < 0.01. **(B)** *In vivo* imaging of H22 tumor-bearing mice administered with DiR-loaded iRGD-NCs and NCs at the time points of 2, 4, 8 and 12 h. **(C)** *Ex vivo* fluorescence imaging of mouse tumors 12 h after the administration of DiR-loaded iRGD-NCs and NCs. **(D**) Distribution of DiR-loaded iRGD-NCs and NCs in mouse tumor tissues 12 h post injection. Red: DiR. Blue: cell nuclei.

The biodistribution of NCs and iRGD-NCs were evaluated in H22 tumor-bearing mice by encapsulating 1,1′-dioctadecyl-3,3,3′,3′-tetramethyl indotricarbocyanine iodide (DiR, a near infrared fluorescence dye) into the nanocarriers. Figure S7B shows the biodistribution of the two nanocarriers in the main organs of tumor-bearing mice. The two nanocarriers were rapidly cleared from the blood and distributed into other organs within 2 h, which was consisted with the results of the pharmacokinetic study. In other organs, the nanocarriers highly accumulated in the heart, liver, spleen, lung and kidney, and the liver and spleen internalized a much greater amount of nanocarriers due to the presence of the reticuloendothelial system. A similar result was also observed in the normal mice (see Supplementary Fig. S7C). Importantly, the accumulation of DiR-loaded iRGD-NCs (iRGD-NCs-DiR) in tumors was greater than the accumulation of DiR-loaded NCs (NCs-DiR) and DiR-loaded PEG2000 modified NCs (PEG-NCs-DiR) (p < 0.05), showing the enhanced performance in tumor targeting. Surprisingly, the accumulation of NCs-DiR in tumor was significantly higher than PEG-NCs-DiR (Fig. 4A), though a number of reports claimed that the PEG coating onto the nanoparticles helped increase the tumor accumulation of drug via enhanced permeability and retention (EPR) effect. It might be because the coating of β-LG (a globular protein) onto the nanomedicine reduced the association with NCs for several serum proteins that might have opsonic activity.

To further confirm the tumor-targeting capacity, *in vivo* fluorescence imaging of tumor-bearing mice injected with NCs-DiR and iRGD-NCs-DiR was performed at 2, 4, 8 and 12 h after injection. The fluorescence intensity of iRGD-NCs-DiR in the tumors was higher than that of NCs-DiR at each time point, and the fluorescence intensity decreased gradually from 2 to 12 h (Fig. 4B), thus suggesting the enhanced accumulation of iRGD-NCs in tumors.

To evaluate the distribution in tumor tissues, the isolated tumors were imaged *ex vivo*, and frozen sections were stained with 4,6-diamino-2-phenyl indole (DAPI) and observed using CLSM. Compared to the NCs-DiR, the iRGD-NCs-DiR group had higher fluorescence intensity (Fig. 4C) in tumors and stronger red signals (Fig. 4D) that indicated the distribution of the nanocarriers in tumor tissue, indicating that the iRGD-NCs-DiR efficiently accumulated and penetrated into the tumor tissue.

### *In vivo* antitumor activity

**Figure 5:**
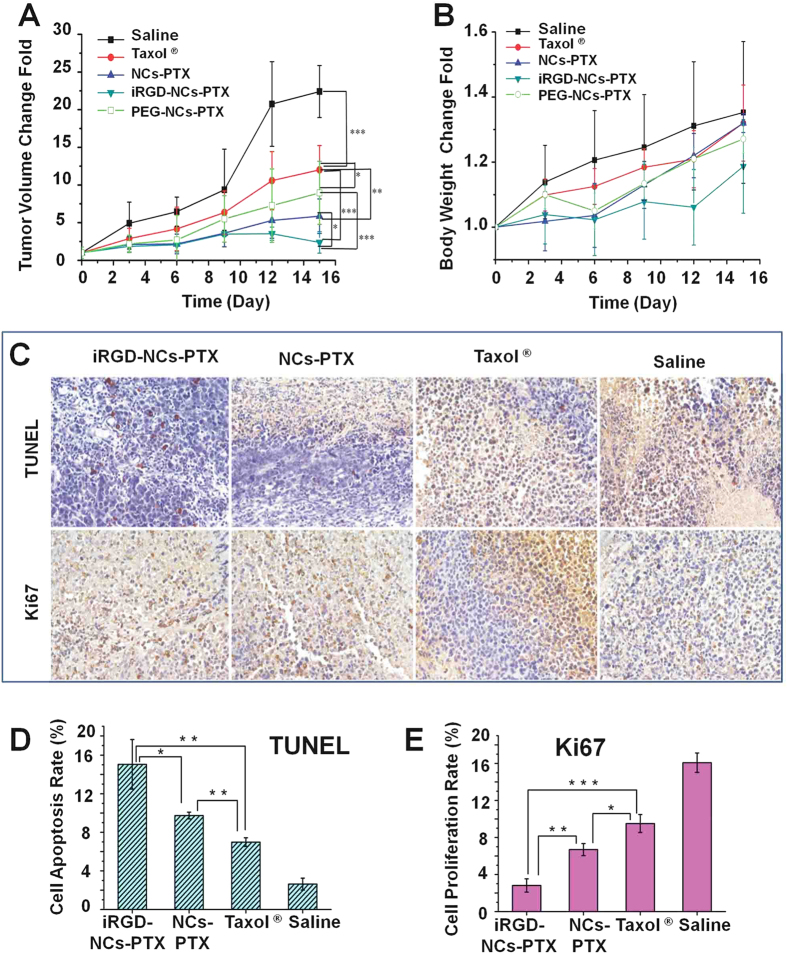
Anti-tumor efficiency. Anti-tumor activity of PTX formulations in H22 tumor-bearing mice (n = 9), after the mice were administered iRGD-NCs-PTX, PEG-NCs-PTX, NC-PTX, Taxol^®^ at the PTX dose of 10 mg/mL, and saline was used as a control. (**A**) Time-related tumor volume in H22 tumor-bearing mice. A comparison of tumor volume between the groups was performed on the 15^th^ day. *p < 0.05, **p < 0.01 and ***p < 0.001. (**B**) Body weight change of the mice after treated with the PTX formulations. (**C**) TUNEL and Ki67 analysis of tumors. The brown-stained cells represent the positive cells. (**D**) Quantitative analysis of cell apoptosis (n = 3). *p < 0.05 and **p < 0.01 indicate groups significantly lower than the iRGD-NCs group. (**E**) Quantitative analysis of cell proliferation (n = 3), *p < 0.05, **p < 0.01 and ***p < 0.001 indicate groups significantly higher than the iRGD-NCs group.

Formulations of saline, Taxol^®^, NCs-PTX and iRGD-NCs-PTX were injected intravenously into H22 tumor-bearing mice via the tail vein. The tumor volumes of each group were recorded, and the change in tumor volume was calculated every three days to evaluate the antitumor activity. As shown in Fig. 5A, the tumors in all groups grew more or less during the study period. The tumors in the saline group grew rapidly without any inhibition, and the tumor volume change fold reached 22.40 ± 3.45 15 days later, while the change folds were 12.01 ± 3.23, 6.98 ± 2.29, 8.95 ± 4.18 and 3.59 ± 1.42 for the groups treated with Taxol^®^, NCs-PTX, PEG-NCs-PTX and iRGD-NCs-PTX, respectively, demonstrating that these formulations inhibited the growth of tumors. The groups treated with NCs-PTX and PEG-NCs-PTX exhibited enhanced tumor growth inhibition compared with Taxol^®^. This difference was ascribed to the high PTX loading and EPR effect, which enhanced the drug bioavailability at the tumor site. Interestingly, the NCs without PEG coating had much stronger tumor suppression effect than PEG-NCs. It was ascribed to the enhanced tumor accumulation from NCs (Fig. 4A). Obviously, iRGD-NCs-PTX suppressed the tumor growth more effectively than that of NCs-PTX and PEG-NCs-PTX, evident by that the tumor volume change fold from the group of iRGD-NCs-PTX was 1.9 and 2.5-fold less than that of NCs-PTX and PEG-NCs-PTX 15 days later, respectively. It was due to that the iRGD improved the accumulation and penetration of the nanocarriers in the tumors.

The body weight change fold of mice after treated with different PTX formulations is shown in Fig. 5B. Compared to other groups, the body weight of group treated with saline rose significantly because of the increase of tumor volume. Among the other groups treated with PTX formulations, the increase of body weight from the iRGD-NCs-PTX was significantly less than that of Taxol 3 days later and that of NCs-PTX and PEG-NCs-PTX after 9 days.

To further test the antitumor activity, cell apoptosis measured using TUNEL and cell proliferation determined with Ki67 *in situ* were evaluated after paraffin sections were prepared from the isolated tumors. The qualitative analysis of TUNEL indicated that the apoptotic cells (brown) from the groups treated with iRGD-NCs-PTX and NCs-PTX were more profound than those from the Taxol^®^ and saline groups (Fig. 5C). Further quantitative analysis revealed that the rates of apoptotic cells were 15.05 ± 2.57%, 9.74 ± 0.35%, 6.98 ± 0.45% and 2.63 ± 0.62% for iRGD-NCs-PTX, NCs-PTX, Taxol^®^ and saline, respectively (Fig. 5D). The Ki67 analysis for cell proliferation (Fig. 5E) further confirmed the results of the TUNEL analysis. It indicated that iRGD-NCs-PTX and NCs-PTX produced better antitumor activity by inducing tumor cell apoptosis and inhibiting tumor cell repopulation. Moreover, the iRGD ligand that homed to the tumor could further enhance the antitumor activity of the NCs-PTX.

### *In vivo* biocompatibility and toxicity

In addition to the tumor, the nanocarriers also distributed to other organs such as the heart, liver, spleen, lung and kidney. Therefore, a study of the *in vivo* biocompatibility of iRGD-NCs and blank NCs was performed. Hematologic analysis and CD68 immunostaining were used to evaluate the inflammatory reaction of mice treated with iRGD-NCs and blank NCs. In the hematologic analysis, the values of important parameters from the groups treated with iRGD-NCs and NCs were not different from the saline group (see Supplementary Table S2). The analysis of CD68 immunostaining is shown in Fig. S8A. The inflammation areas in the iRGD-NCs and NCs groups in each organ (heart, liver, spleen, lung and kidney) were similar to those of the saline group. Although the biodistribution study indicated that the nanocarriers were also distributed to other organs, iRGD-NCs and NCs did not cause significant inflammation in these organs, thereby suggesting better biocompatibility.

To further evaluate the toxicity of the iRGD-NCs and NCs, mice were treated with iRGD-NCs and NCs loaded with PTX, and sections of various organs were prepared. The analysis of the histopathology indicated that there was no lymphocytic infiltration, microgranulation or degenerative necrosis of hepatocytes and splenocytes in the two groups. Tissues from the heart, liver, spleen, lung and kidney also showed no remarkable changes in morphology compared with the control (see Supplementary Fig. S8B). Therefore, the present nanocarriers did not produce toxicity to other organs.

## Discussion

Low drug payload, rapid blood clearance and premature burst release are inherent drawbacks of conventional nanoparticles, owing to poor tumor selectivity and side effects in other tissues^19^. Core-shell structured nanocarriers with high drug-loading, leading to a higher deposition of drug into cancer cells upon delivery, are especially promising for cancer therapy^37^ because they could largely protect the healthy organs from the toxic drugs, prevent the decomposition/denaturing of the drugs before reaching the target site and minimize the side effects^38^. Herein, we present a nanocarrier with a core-shell structure. Compared with the conventional nanomedicines, the drug loading of NCs for anticancer drugs with low water solubility was more than 12%, indicating a high drug payload. In theory, a nanocarrier with this characteristic would produce better therapeutic effects against cancer. Hence, the *in vitro* and *in vivo* anticancer activity was evaluated.

As expected, the NCs-PTX had a higher cytotoxicity against cancer cells than Taxol^®^ (commercial product); additionally, the iRGD-NCs further improved the cytotoxicity due to the enhanced cellular uptake of the NCs through iRGD homing to α_v_β_3_ integrin. It was evident that the percentages of apoptosis in H22 cells induced by the iRGD-NCs-PTX and NCs-PTX were about 5 and 3-fold greater than that induced by Taxol^®^ (Fig. S6D), which might be due to that the nanocarriers with high loading had potential to overcome the tumor drug resistance^39^.

We demonstrated that significant enhancement in pharmacokinetics of PTX was achieved by PTX-NCs in comparison with Taxol^®^, exhibited as markedly prolonged MRT and increased AUC. As shown in Table 1, the clearance (CL) values for the NCs and iRGD-NCs were approximately 14 and 21-fold less than that of Taxol^®^, confirming our assertion. Moreover, the values of CL and MRT from PEG-NCs was significantly less than that from NCs, further verifying the enhanced prolonged circulation effect from NCs. As well known, serum albumin can prevent nanoparticles from being recognized and decreased phagocytic clearance, thereby leading to extending blood circulation and enhanced delivery of nanoparticles^40^,^41^,^42^. β-LG had a similar structure with serum albumin, thus it would behave similarly to the serum albumin *in vivo*, which its coating onto nanoscaled oil-cores would inhibit phagocytic clearance. In fact, it was recently reported that the coating of globulin like CD47 onto nanocarriers prolonged the circulation time of nanoparticles by more than 10-fold in contrast with PEG coating^43^,^44^. Secondly, the hydrophilic shell of the NCs, which reduced nanoparticle association with opsonin, could increase nanocarrier stability and prolong the plasma half-life by preventing rapid renal clearance and uptake by the reticuloendothelial system^45^,^46^. Therefore, we concluded that the coating of β-LG play acritical role in enhancement in pharmacokinetics of PTX.

Significant tumor growth inhibition with good biocompatibility was seen in the groups treated with the NCs-PTX and iRGD-NCs-PTX compared to the group treated with Taxol^®^. First, the nanocarriers with high drug loading increased the drug bioavailability at the sites of action^19^. A comparison of the AUCs between the NCs and Taxol^®^ groups indicated that the NCs significantly increased bioavailability (Table 1), thereby suggesting that the NCs could deliver a considerable amount of drug to the target sites. Second, the sustained release profile of the nanocarriers with a lipid core prolonged the drug action because the NCs could continuously release the drug over a long time frame (Fig. S4)^47^. Third, prolonged particle circulation in blood improved the EPR effect. Fourth, the homing effect of iRGD to integrins brought the NCs to the tumor tissues in an actively targeted way, which was evident in that the accumulation of iRGD-NCs in the tumors was significantly increased and the penetration into the tumors was also improved (Fig. S6E). Additionally, no toxic materials were used to create the NCs, thereby ensuring better *in vitro* and *in vivo* biocompatibility from these nanocarriers.

Understanding the mechanism of nanomedicine endocytosis is very important for controlling activity and toxicity. The cellular uptake of iRGD-NCs was significantly inhibited by M-β-CD and NaN_3_ with deoxyglucose (Fig. 2D), indicating the internalization of the iRGD-NCs was energy- and cholesterol-dependent. M-β-CD depletes cholesterol from the cell membrane, and cholesterol plays an important role in macropinocytosis, caveolin-mediated endocytosis and lipid-raft-mediated endocytosis^48^. Additionally, the lack of an effect on endocytosis with Cyto-D and nystatin treatment suggested that macropinocytosis and caveolin-mediated endocytosis were not involved. Hence, the endocytosis of iRGD-NCs was mediated by lipid-rafts. Moreover, the uptake was inhibited by the combination of NaN_3_ and deoxyglucose (energy-depletion agents), which indicated that the internalization mediated by iRGD is an energy-dependent pathway^49^. However, the internalization of NCs was only inhibited by the M-β-CD; it therefore suggested that the endocytosic mechanism be altered by the ligand. Recently, it was also reported that uptake pathway of the nanomedicines could be changed by the conjugation of ligands^50^.

In summary, NCs and NCs with iRGD-modification with a high drug payload for anticancer drugs were successfully prepared. These NCs could significantly inhibit tumor growth; and the antitumor activities could be further improved by iRGD modification because enhancing the accumulation and penetration into the tumor was achieved. Importantly, significant improvement in pharmacokinetics of PTX was achieved by PTX-NCs in comparison with Taxol^®^, exhibited as markedly prolonging circulation in blood and increased bioavailability. To our knowledge, few reports indicated that the protein-coated nanoscaled oil-cores could change the pharmacokinetics of PTX significantly. The endocytosis of the nanocarriers was mediated by lipid rafts in an energy-dependent manner, leading to better cytotoxicity of PTX against cancer cells. In conclusion, the present nanocarriers with high drug-loading capacity were proven to be an efficient tumor-targeting drug delivery system and have potential for medical applications.

## Method

Please refer to the Supplemental Information for the other Methods.

### Cellular uptake, internalization mechanism and intracellular tracking

A549 cells (Nanjin Key GEN Biotech, Nanjing, China) were seeded on 24-well plates at a density of 5 × 10^4^ cells/well and cultured for 24 h. Then, the cells were incubated with C-6-loaded iRGD-NCs and NCs in fresh RPMI-1640 (Thermo Fisher Scientific Inc, MA, USA) at a C-6 concentration ranging from 80 to 1000 ng/mL. After washing 3 times with PBS and digesting with 0.25% trypsin buffer (Thermo Fisher Scientific Inc), the cells were observed using an inverted fluorescence microscope (Nikon Eclipse Ti-s, Japan). The fluorescent intensity was measured using a FCM (BD FACSCalibur, USA).

The cells were seeded on 6-well plates at a density of 1 × 10^5^ cells/well and cultured for 24 h at 37 °C. Then, the culture medium was replaced with fresh medium that contained endocytic pathway inhibitors, including Cyto-D (10 μg/mL), nystatin (10 μM), Cpz (10 μg/mL), nocodazole (20 μM), M-β-CD (2.5 mM), monensin (200 nM) and NaN_3_ (10 mM) with DG (50 mM). After being saturated with the inhibitors for 30 min at 37 °C, the cells were incubated with iRGD-NCs-C-6 and NCs-C-6 for 2 h. The fluorescence intensity of the cells was measured using FCM after digested from the plates.

A549 cells were seeded on 30 mm glass bottom cell culture dishes at a density of 1 × 10^5^ cells/dish and cultured for 24 h. Then, the cells were incubated with C-6-loaded iRGD-NCs and NCs in fresh RPMI-1640 at a C-6 concentration of 400 ng/mL for 2 h. After being washed 3 times with PBS, the cells were incubated in medium containing Lyso-Tracker Red for 1 h. Then the cells were fixed with 4% paraformaldehyde after washing, and stained by DAPI (Beyotime Institute of Biotechnology, China) for 15 min. Finally, the cells were observed using confocal laser scanning microscopy (LSM700, Zeiss, Germany).

### *In vivo* tumor targeting

The animals used in the experiments received care in compliance with the Principles of Laboratory Animal Care and the Guide for the Care and Use of Laboratory Animals. All the animal experiments were performed in accordance with the protocol approved by the China Pharmaceutical University Institutional Animal Care and Use Committee.

The mice were anesthetized with a 4% novochlorhydrate solution, and fluorescence images were taken using an *in vivo* imaging system at the time points of 2, 4, 8 and 12 h after intravenous administration via the tail vein at a dose of 0.5 mg/kg DiR. At 12 h post injection, the mice were sacrificed, and then the tumors were isolated and frozen at −80 °C. Finally, the frozen tumors were prepared, sectioned, stained with DAPI for 5 min and observed using CLSM.

### *In vivo* antitumor activity

The antitumor activity of iRGD-NCs-PTX, PEG-NCs-PTX, NCs-PTX, Taxol^®^ and saline was evaluated by administering them to the H22 tumor-bearing mice via the tail vein at a dose of 10 mg/kg PTX (n = 9)^1^. About 200 μL of PTX formulation was administered to the mice every three days, and the change fold of the tumor volume was calculated every three days.

To determine the number of apoptotic cells using TUNEL method, the mice were sacrificed 15 days after treatment, and the tumors were isolated. The isolated tumors were fixed in 4% paraformaldehyde, embedded in paraffin and cut into 5 μm sections. After deparaffinization and rehydration, the sections were incubated in a 0.1% sodium citrate solution containing 0.1% Triton X-100 for 15 min on ice, washed 3 times with PBS and incubated with 3% H_2_O_2_ for 15 min at room temperature to block endogenous peroxidase activity. Then, the sections were incubated with a mixture of terminal deoxynucleotidyl transferase (TDT) in a humidified chamber at 37 °C for 60 min, followed by a PBS wash and incubation with peroxidase-conjugated streptavidin in a humidified chamber for 30 min at room temperature. After being rinsed with PBS, the sections were incubated with two drops of diaminobenzidine (DAB) solution and counterstained with hematoxylin staining solution for 10 min. Finally, an image was taken using an optical microscope after the sections were dehydrated with ethanol (95%) and mounted with neutral balsam. The extent of apoptosis was evaluated by counting the TUNEL-positive cells (brown stained) as well as the total number of cells^51^. The proliferative cells in the tumors were measured with Ki67. After deparaffinization and rehydration, the sections of isolated tumor were heated in boiling antigen retrieval buffers for 20 min and cooled in water. The sections were incubated with Ki67 antibodies (Wuhan Boster Biological Technology, Wuhan, China) and IgG-Fab’-HRP polymers for 30 min and then incubated with 3% H_2_O_2_ for 15 min, rinsed 3 times with PBS and incubated with 100 μL goat serum for 20 min. Subsequently, the sections were incubated with 2 drops of DAB solution, counterstained with hematoxylin staining solution for 10 min, dehydrated with ethanol (95%) and mounted with neutral balsam. Finally, the sections were observed and imaged using an optical microscope. The cell proliferation rate was also assessed using a method similar to a TUNEL assay.

### Statistical analysis

The data were presented as the mean ± SD. The statistical significance between the groups was determined using a one-way ANOVA analysis. p < 0.05 was considered to be a significant difference.

## Additional Information

**How to cite this article**: Jin, Z. *et al.* Core-shell nanocarriers with high paclitaxel loading for passive and active targeting. *Sci. Rep.* **6**, 27559; doi: 10.1038/srep27559 (2016).

## Supplementary Material

Supplementary Information

## Figures and Tables

**Table 1:** Pharmacokinetic parameters of PTX after intravenous injection of iRGD-NCs-PTX, PEG-NCs-PTX and NCs-PTX at a dose of 10 mg/kg in rats (n = 3).

Formulations	AUC_0-∞_(mg·h/L)	MRT (h)	k (h^−1^)	CL (L·h^−1^)
Taxol^®^	4.38 ± 3.57	1.78 ± 0.88	2.99 ± 2.88	1.68 ± 1.19
NCs-PTX	18.84 ± 7.07	15.17 ± 6.15	0.044 ± 0.023	0.12 ± 0.054
PEG-NCs-PTX	5.67 ± 0.60	3.03 ± 1.78	0.99 ± 0.69	0.19 ± 0.089
iRGD-NCs-PTX	25.61 ± 3.98	6.52 ± 1.23	0.098 ± 0.0093	0.08 ± 0.013

k: elimination rate constant.
