# Supplementary material for: Core-shell nanocarriers with high paclitaxel loading for passive and active targeting
Source: Sci Rep. 2016 Jun 9;6:27559. doi: 10.1038/srep27559 (PMC4899770; doi:10.1038/srep27559)
Supplement: Supplementary Information [file srep27559-s1.doc]

**Supporting Information**

**Core-shell nanocarriers with high** **paclitaxel loading for passive and active targeting**

Zhu Jin^1^, YaqiLv^1^, Hui Cao, Jing Yao, Jianping Zhou, Wei He***, and Lifang Yin***

Department of Pharmaceutics, School of Pharmacy, China Pharmaceutical University, Nanjing, 210009, PR China

*****Correspondence and requests for materials should be addressed to He, W or Yin, LF (email: weihe@cpu.edu.cn, lifangyin_@163.com)

^1^These authors contributed equally to this work.

**Methods**

**Preparation and characterization of NCs and iRGD-NCs.** NCs were prepared using a method similar to our previous report[^1^](#_ENREF_1). PTX (45 mg, Yunnan Hande Bio-Tech, Kunming, China) and lecithin (50 mg, Sinopharm Chemical Reagent, Shanghai, China) were dissolved in 2 mL oil phase (LABRAFIL M CS1944) (Gattefossé, Cedex, France) using an ultrasonic dispersion method. Then, the oil phase was added to 30 mL aqueous solution containing 1% β-LG (w/w) (Sigma-Aldrich, MO, USA), which was denatured at 85 °C for 30 min and adjusted to pH 8.5. Subsequently, the mixture was dispersed at 10,000 rpm using a high-speed disperser (Ningbo Scientz Biotechnology Co. Ltd., China) and homogenized at 700 bar for 30 cycles using an AH-2010 high pressure homogenizer **(ATS Engineering Inc., Canada)**. NCs-PTX, NCs-C-6 and NCs-DiR were prepared with the same procedure except that PTX (45 mg), C-6 (1.5 mg, Sigma-Aldrich, MO, USA) and DiR (4 mg, Biotium, CA, USA) were dissolved in 2 mL of LABRAFIL M CS1944 in advance.

iRGD (4 mg, GL Biochem Ltd., Shanghai, China) and MAL-PEG2000-NHS (7 mg, Jenkem technology, Beijing, China) were reacted in 1 mL hanks balanced salt (HBS) solution (20 mM HEPES, 0.15 M NaCl, pH 7.1) in a N_2_ atmosphere at 4 °C for 24 h. Then, the dispersed suspension of NCs was added to the reacted solution and reacted for 1 h at pH 8.5. Finally, the iRGD-NCs were purified using a Sepharose® CL-4B column[^2-4^](#_ENREF_2). PEG-NCs were prepared by modifying mPEG2000-NHS (Jenkem technology) to NCs directly at pH 8.5 for 1 h.

Particle size and size distribution were measured via the DLS method using a ZetaPlus Zeta Potential Analyzer (Brookhaven Instruments, New York, USA). The samples were diluted adequately before measurement.

The morphology of the iRGD-NCs was observed using a JEM-1230 TEM (Tokyo, Japan). One drop of diluted iRGD-NCs was placed on a copper grid and dried at room temperature. TEM photographs were taken at 200 kV after the iRGD-NCs were stained with 2 % phosphotungstic acid.

The surface of the iRGD-NCs was examined using a Philips XL30 SEM (Eindhoven, Holland). The suspension of iRGD-NCs was deposited on a brass stub and dried at room temperature. After the iRGD-NCs were gold-coated for 200 s using a sputter coater, the photographs were taken at an excitation voltage of 10 kV.

The surface morphology of the iRGD-NCs was measured using an AFM (Veecodi Nano Scope V, USA). One drop of diluted iRGD-NCs was placed on the surface of a clean silicon wafer and dried at room temperature. Then, the sample was observed using the AFM with a 5 μm scanner in contact mode.

The purified PTX-loaded NCs and iRGD-NCs were incubated with 5 % pepsin for 12 h to digest the β-LG and break the NCs. The digestion solution was diluted with methanol and centrifuged for 10 min at 10,625 g, and the supernatant was analyzed using a high performance liquid chromatography (HPLC) system (SHIMAZU LC-10AT, Kyoto, Japan). The EE and DL were calculated using the following formulas:

EE (%) = Amount of PTX encapsulated/Amount of PTX added × 100 %

DL (%) = Amount of PTX encapsulated/Amount of β-LG × 100 %

**Validation of the connection between iRGD and NCs.** A colorimetric method that could verify the presence of PEG linker was used to confirm the conjugation between iRGD-PEG2000-NHS and the NCs indirectly[^5^](#_ENREF_5). Briefly, purified iRGD-NCs solution (1 mL) was incubated with 5% trypsin solution (0.15 mL) at 37 °C for 12 h and then stirred vigorously with a mixture of ammonium ferrothiocyanate solution (2 mL) and trichloromethane (2 mL) for 30 min. After centrifugation, the trichloromethane supernatant was retained, and its absorbance was measured using an ultraviolet spectrophotometer. In addition, unmodified NCs were tested in the same way as a control.

**In vitro drug release.** The drug release from PTX formulations was carried out by a dialysis method and conducted in a ZRS-8G release tester (Tianjin, China)[^6^](#_ENREF_6). Briefly, 0.5 mL of the PTX formulations was added into a dialysis bag (MWCO=8000–10000 Da). Then, the bags were fixed on the paddle of the release tester and immersed in a beaker with 100 mL of sodium salicylate-phosphate buffer saline (PBS) solution (1 M, pH 7.4) and kept at 37 °C with a paddle revolution speed of 50 rpm. At specific time points, 0.5 mL of sample was withdrawn and centrifuged at 10,000 g for 10 min. The drug content in the samples was assayed using an HPLC method.

The PTX determination was carried out with an HPLC system (Shimadazu, Tokyo, Japan) consisted of a LC-10AT pump and a SPD10A UV–Vis detector (Shimadazu, Tokyo, Japan). The separation was performed on a on a Diamonsil C_18_ column (4.6 mm × 250 mm) at 227 nm. The mobile phase was a mixture of acetonitrile and water (65: 35, v/v) that was pumped at a rate of 1.0 mL/min at 40 °C. The injection volume for analysis was 20 μL.

**In vitro cytotoxicity.** To assess the cytotoxicity, the cell viabilities against blank NCs and iRGD-NCs were investigated. A549, MDA-MB-435, SGC-7901 and H22 cells (Nanjin KeyGEN Biotech) were seeded on 96-well plates at a density of 5×10^3^ cells/well and cultured for 24 h. These four types of cells were then incubated with NCs or blank iRGD-NCs with β-LG at various concentrations. H22 cells were also exposed to iRGD-NCs-PTX, NCs-PTX or Taxol® at various concentrations for 48 h. Cell viability was evaluated using an 3-(4,5-dimethylthiazol-2yl)-2,5-diphenyltetrazolium bromide (MTT) assay, and IC_50_ values were calculated using the OriginPro software (OriginLab Co., MA, USA).

**Cell apoptosis assay.** H22 cells were seeded on 12-well plates at a density of 1×10^5^ cells/well and cultured for 24 h. The cells were then incubated with iRGD-NCs-PTX, NCs-PTX or Taxol® for 24 h at a PTX concentration of 5,000 ng/mL. The cell apoptosis was detected with an FCM analysis using an Annexin V-FITC/PI apoptosis detection kit (Bender Medsystems, Vienna, Austria).

**Tumor spheroid penetration.** Two hundred microliters of 2% low-melting-temperature agarose was added to each well of a 48-well plate to coat the bottom and prevent cell adhesion. Then, A549 cells were seeded in each well at a density of 2×10^3^ cells/well and cultured at 37 °C for 7 days. C-6-loaded NCs and iRGD-NCs were added and incubated with the tumor spheroids at 37°C for 4 h. Finally, the tumor spheroids were washed with PBS and observed using CLSM.

**Pharmacokinetics in rats.** The animals used in the experiments received care in compliance with the Principles of Laboratory Animal Care and the Guide for the Care and Use of Laboratory Animals. All the animal experiments were performed in accordance with the protocol approved by the China Pharmaceutical University Institutional Animal Care and Use Committee.

The SD rats (male, 180–220 g) were randomly divided into three groups (n=4), which were intravenously injected with Taxol®, NCs-PTX, PEG-NCs-PTX or iRGD-NCs-PTX at a dose of 10 mg/kg PTX. Blood samples (0.5 mL) were collected from rat orbit at the time points of 0.083, 0.25, 0.5, 1, 2, 4, 6 and 8 h. The blood samples were centrifuged for 10 min at 5,000 rpm in a desktop centrifuge (Anke TGL-16G, Shanghai, China), and the plasma was separated and transferred to microcentrifuge tubes. The PTX in plasma was measured using an HPLC system after extraction with ethylether and redissolved in methanol. The pharmacokinetic parameters of PTX were calculated using a non-compartmental model with Kinetica 5.0 (Thermo Fisher Scientific Inc., MA, USA).

Two hundred microliter of plasma samples were mixed with 10 µL of internal standard (100 ng/mL diazepam in methanol), followed by vortexing for 60 s. Following mixing into 800 µL of ethylether, the sample was vortexed for 2 min and centrifuged at 12,700 g for 10 min. Subsequently, the supernatant was collected and dried under a stream of nitrogen. The residual was redisolved in 100 µL of mobile phase, votexed for 3 min and centrifuged at 10,625 g for 5 min[^7^](#_ENREF_7). Finally, 20 µL of the supernatant was injected into the HPLC system for analysis under the conditions described below.

PTX measurement was performed on a LC-10AT HPLC system (Shimadzu, Japan). It was separated at 30 °C using an ODS-C18 column (4.6 mm × 250 mm, Diamonsil, China). The samples were eluted with acetonitrile and water (54: 46, v/v) at the flow rate of 1.0 mL/min and monitored at 227 nm[^8^](#_ENREF_8). The regression equation used for the plasma concentration determination was y = 0.0244x + 0.0003 (R^2^ = 0.9999), which resulted in linear concentrations from 0.2–500 µg/mL. The absolute recoveries of low (2 µg/mL), medium (20 µg/mL) and high (200 µg/mL) quality control levels were 98.9%, 97.9%, and 102.4%, respectively, and the intra-day and inter-day precision were not greater than 15%.

***In vivo* biodistribution.** H22 tumor-bearing mice were prepared as follows: a cell suspension (0.2 mL) with a density of 1×10^7^ cells/mL was injected subcutaneously into the scapula region of the ICR mice (male, 18-22 g). Treatment began when the tumors reached a volume of 10×10×5 mm^3^.

The H22 tumor-bearing mouse groups receiving DiR-loaded iRGD-NCs, PEG-NCs or NCs were treated intravenously via the tail vein at a dose of 0.5 mg/kg DiR (n=5). At the time points of 2, 4 and 8 h post injection, the mice were sacrificed and dissected to collect blood and other major tissues (hearts, livers, spleens, lungs and kidneys). The mean fluorescence intensity of the collected samples was measured using an *in vivo* imaging system (In-Vivo FX PRO, Carestream, Canada). Normal male mice also underwent the same procedure.

***In vivo* biocompatibility and toxicity.** Male ICR mice were randomly divided into groups receiving either saline, blank iRGD-NCs or blank NCs, which were injected intravenously via the tail vein at the time points of 1, 4, 7 and 11 days. Twelve days post-administration, the mice were sacrificed and both the blood and major tissues (heart, liver, spleens, lung and kidney) were harvested. The blood samples were analyzed using a blood-cell counter. The other major tissues were subjected to CD68 immunohistochemical and histopathology analysis. The paraffin sections (5 µm) of heart, liver, spleen, lung and kidney were prepared for the CD68 assay. Then, the sections were, in sequence, heated in boiling antigen retrieval buffers for 20 min, cooled in water, incubated with 3% H_2_O_2_ for 15 min, rinsed with PBS 3 times, incubated with 100 µL goat serum for 20 min, treated with 100 µL CD68 antibodies (Wuhan Boster Biological Technology)at 37 °C for 2 h, washed with PBS 3 times, cultured with 100 µL IgG-Fab’-HRP polymers at 37 °C for 30 min, washed with PBS, incubated with two drops of DAB solution, counterstained with hematoxylin staining solution for 10 min, dehydrated with ethanol (95%) and mounted with neutral balsam. Finally, the sections were viewed and images were obtained using an optical microscope.

The formulations of saline, Taxol®, NCs-PTX and iRGD-NCs-PTX were also injected intravenously via the tail vein at the time points of 1, 4, 7 and 11 days at a dose of 10 mg/kg PTX. The major tissues (heart, liver, spleens, lung and kidney) were isolated on the 12th day after administration, and 5µm thick tissue sections were subsequently prepared. The sections were observed and imaged using an optical microscope after being stained with hematoxylin staining solution for 10 min, rinsed with distilled water for 10 min, washed with ethanol (95%) for 5 s, stained with eosin staining solution for 2 min, washed with ethanol (95%) two times, dehydrated with ethanol (95%) and mounted with neutral balsam.

**References**

1. He, W. *et al.* Nanoemulsion-templated shell-crosslinked nanocapsules as drug delivery systems. *Int J Pharm* **445**, 69-78(2013).

2. Zhu, S. *et al.* RGD-Modified PEG-PAMAM-DOX Conjugate: In vitro and in vivo targeting to both tumor neovascular endothelial cells and tumor cells. *Adv Mater* **23**, 84-89(2011).

3. Zhang, L. *et al.* RGD-modified PEG–PAMAM–DOX conjugates: In vitro and in vivo studies for glioma. *Eur J Pharm Biopharm* **79**, 232-240 (2011).

4. Wang, K. *et al.* Tumor penetrability and anti-angiogenesis using iRGD-mediated delivery of doxorubicin-polymer conjugates. *Biomaterials* **35**, 8735-8747, (2014).

5. Nag, A., Mitra, G. & Ghosh, P. C. A colorimetric assay for estimation of polyethylene glycol and polyethylene glycolated protein using ammonium ferrothiocyanate. *Anal Biochem* **237**, 224-231 (1996).

6. He, W. *et al.* Food protein-stabilized nanoemulsions as potential delivery systems for poorly water-soluble drugs: preparation, in vitro characterization, and pharmacokinetics in rats. *Int J Nanomedicine* **6**, 521-533 (2011).

7. Zhang, L., He, Y., Ma, G., Song, C. & Sun, H. Paclitaxel-loaded polymeric micelles based on poly(ɛ-caprolactone)-poly(ethylene glycol)-poly(ɛ-caprolactone) triblock copolymers: in vitro and in vivo evaluation. *Nanomedicine: NBM* **8**, 925-934 (2012).

8. Milane, L., Duan, Z.-f. & Amiji, M. Pharmacokinetics and biodistribution of lonidamine/paclitaxel loaded, EGFR-targeted nanoparticles in an orthotopic animal model of multi-drug resistant breast cancer. *Nanomedicine: NBM* **7**, 435-444 (2011).

**Table S1.** Physicochemical characterization of NCs-PTX and iRGD-NCs-PTX (n=3)

| **Groups** | **Particle size (nm)** | **PDI** | **Zeta Potential**  **(mV)** | **EE (%)** | **DL (%)** |
| --- | --- | --- | --- | --- | --- |
| NCs-PTX | 181.7±2.60 | 0.086±0.030 | –31.38±0.35 | 86.04±1.29 | 12.91±0.19 |
| iRGD-NCs-PTX | 196.3±3.30 | 0.138±0.010 | –28.63±0.76 | 83.18±2.44 | 12.48±0.37 |

**Table S2.** Hematologic analysis of mice after four *i.v.* injections of saline, blank iRGD-NCs and blank NCs (n=3)

| **Groups** | **RBC (10^12^/L)** | **HCT (%)** | **MCV (fL)** | **PLT (10^9^/L)** | **WBC (10^9^/L)** | **HGB (g/L)** |
| --- | --- | --- | --- | --- | --- | --- |
| Saline | 3.16±0.57 | 16.20±0.99 | 16.20±0.99 | 2803.00±131.50 | 5.27±2.35 | 80.00±11.90 |
| NCs | 3.20±0.65 | 18.30±3.67 | 57.40±0.29 | 2663.00±140.00 | 5.20±0.96 | 81.00±9.90 |
| iRGD-NCs | 3.44±0.30 | 19.80±1.78 | 57.70±0.00 | 3071.00±709.20 | 6.45±1.06 | 87.00±8.50 |


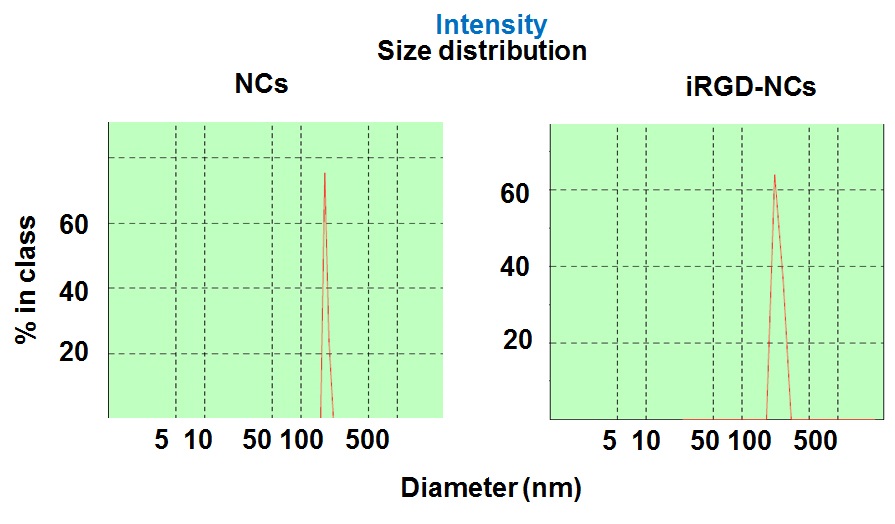


**Figure S1.** Particle size and size distribution of NCs and iRGD-NCs.


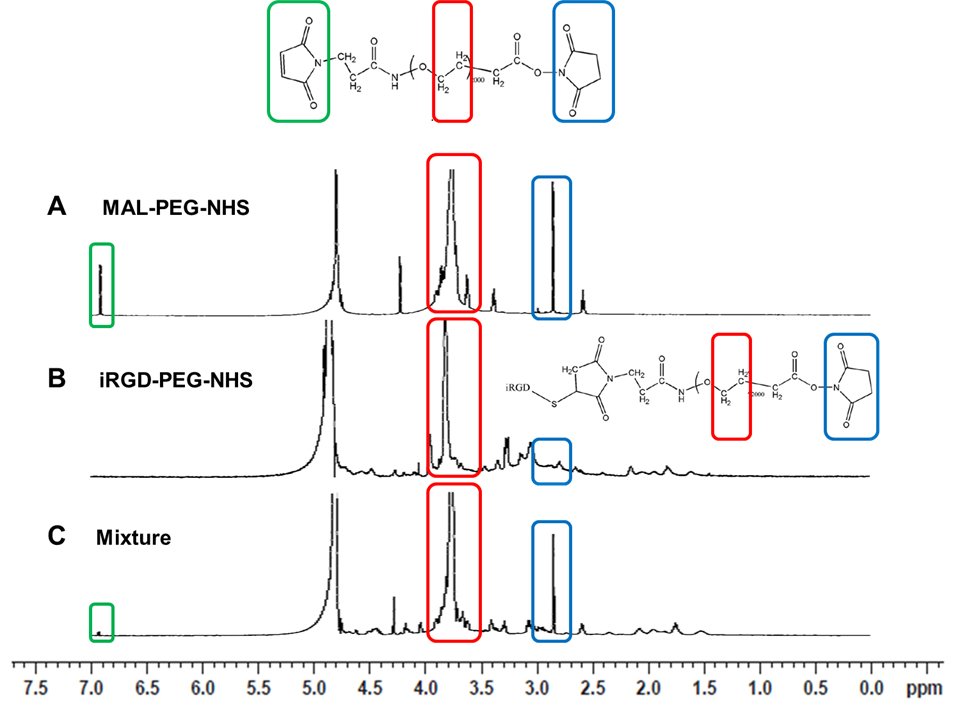


**Figure S2.** ^1^H-NMR spectrum of **(A)** MAL-PEG2000-NHS, **(B)** iRGD-PEG2000-NHS and **(C)** physical mixture of iRGD and MAL-PEG2000-NHS.

**
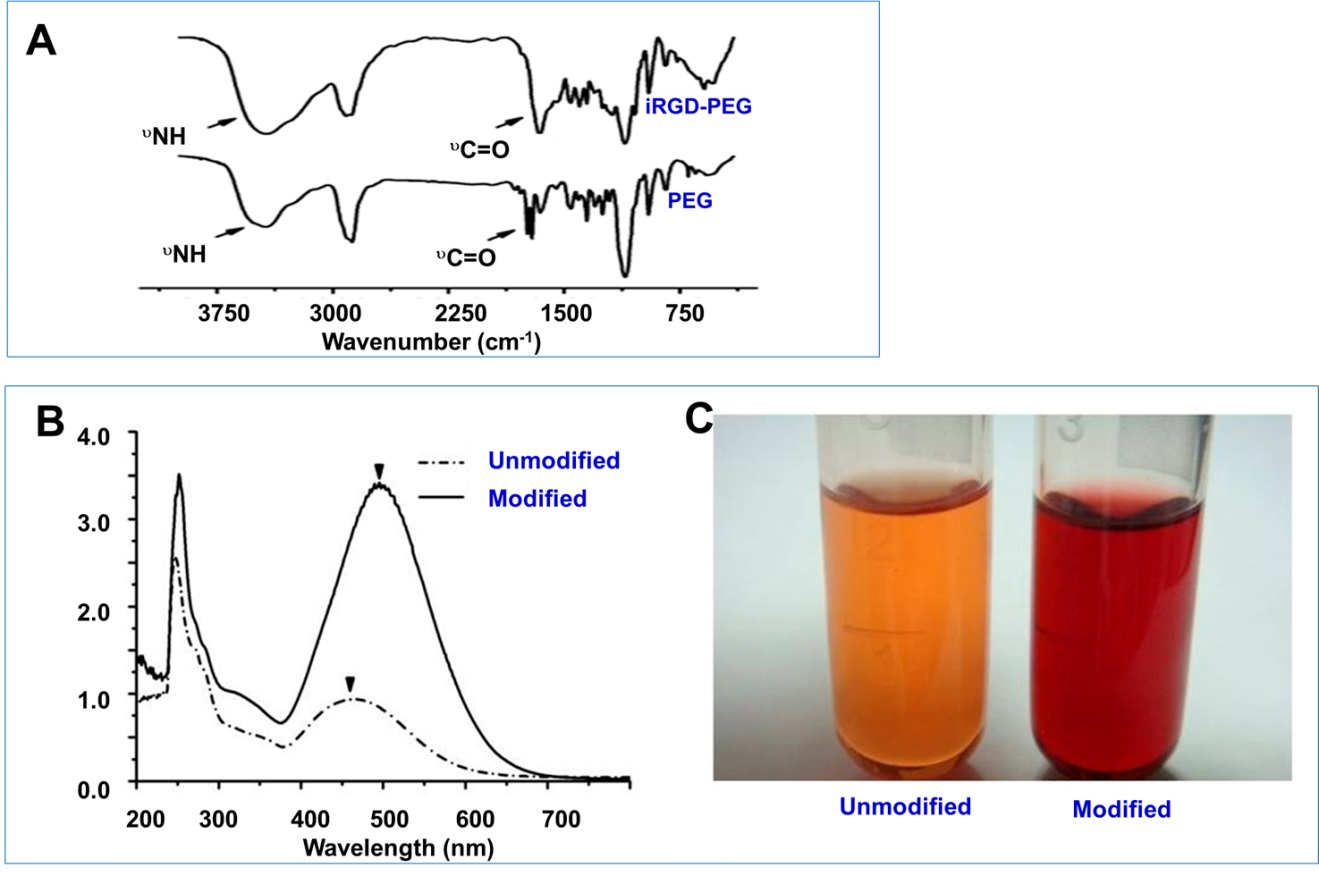
**

**Figure S3. (A)** IR spectrum of iRGD-PEG2000-NHS and MAL-PEG2000-NHS. **(B)** UV-Vis spectrum and **(C)** colour reaction of iRGD-PEG2000-NHS modified and unmodified NCs.


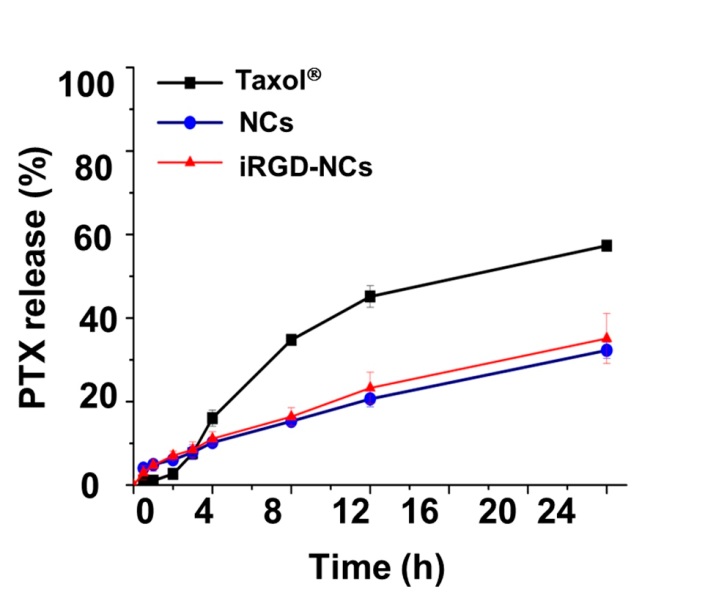


**Figure S4.** *In vitro* drug release from PTX formulations.

**
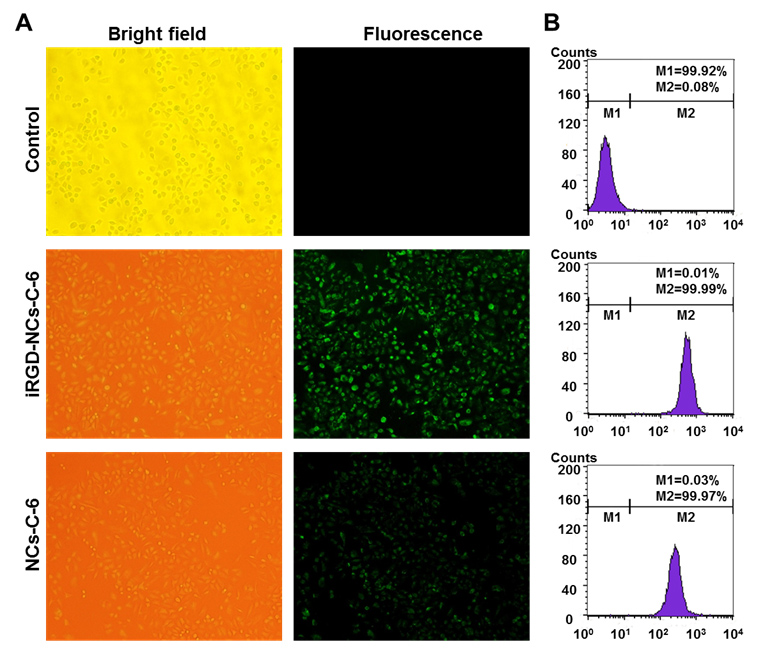
**

**Figure S5.** Cellular uptake of 400 ng/mL C-6 loaded iRGD-NCs or NCs. **(A)** Fluorescence images (Green: C-6) and **(B)** quantitative analysis.


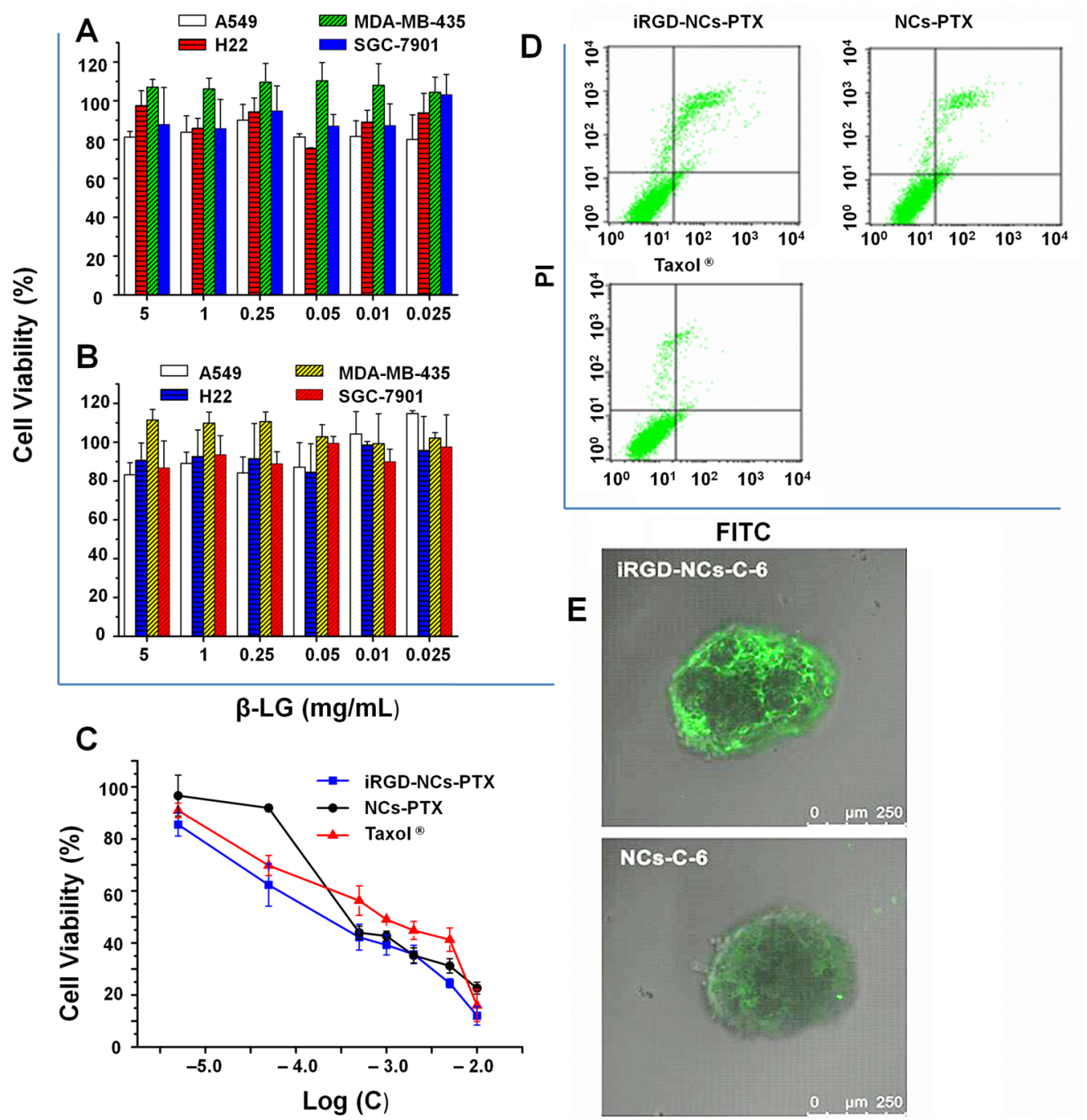


**Figure S6. (A**, **B)** *In vitro* cytotoxicity of blank NCs and iRGD-NCs. **(C)** Anti-proliferation activity of PTX formulations in H22 cells. The cells were incubated with iRGD-NCs-PTX, NCs-PTX and Taxol® at various PTX concentrations for 48h (n=5). **(D)** H22 cell apoptosis induced by PTX formulations. The cells were treated with the formulations at a PTX dose of 5 μg/mL for 24 h. **(E)** Penetration of iRGD-NCs or NCs in A549 tumor spheroids. The tumor spheroids were treated with 400 ng/mL C-6-loaded iRGD-NCs and NCs for 4 h.


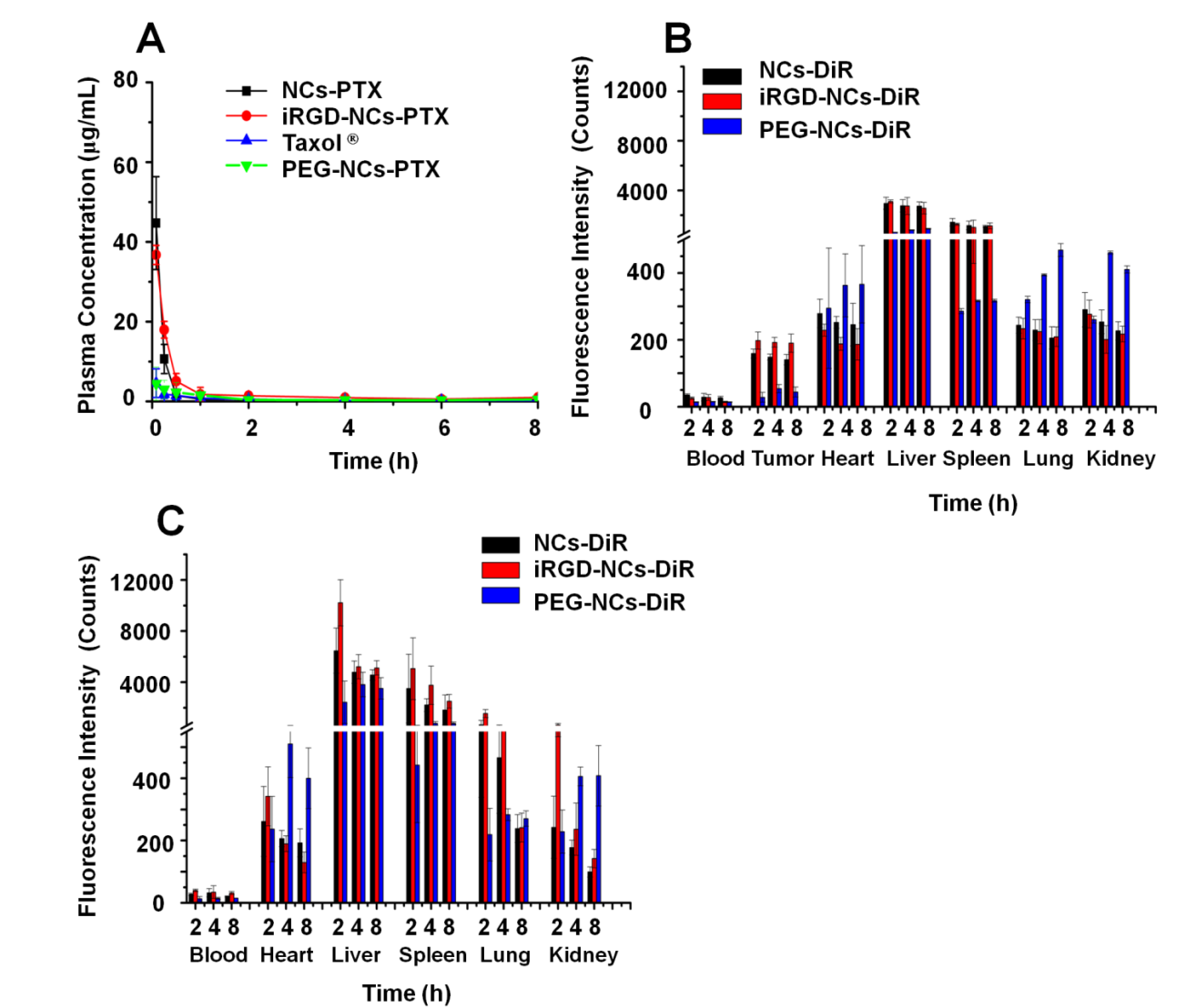


**Figure S7. (A)** Pharmacokinetic profiles of rats administered PTX-loaded iRGD-NCs, PEG-NCs and NCs via the tail vein at a dose of 10 mg/kg of PTX (n=3). **(B, C)** *In vivo* distribution of DiR-loaded iRGD-NCs, PEG-NCs and NCs in H22 tumor-bearing mice and normal mice at 2, 4 and 8 h after administration. The fluorescence intensity represents the quantity of iRGD-NCs, PEG-NCs and NCs in the tissues (n=5).


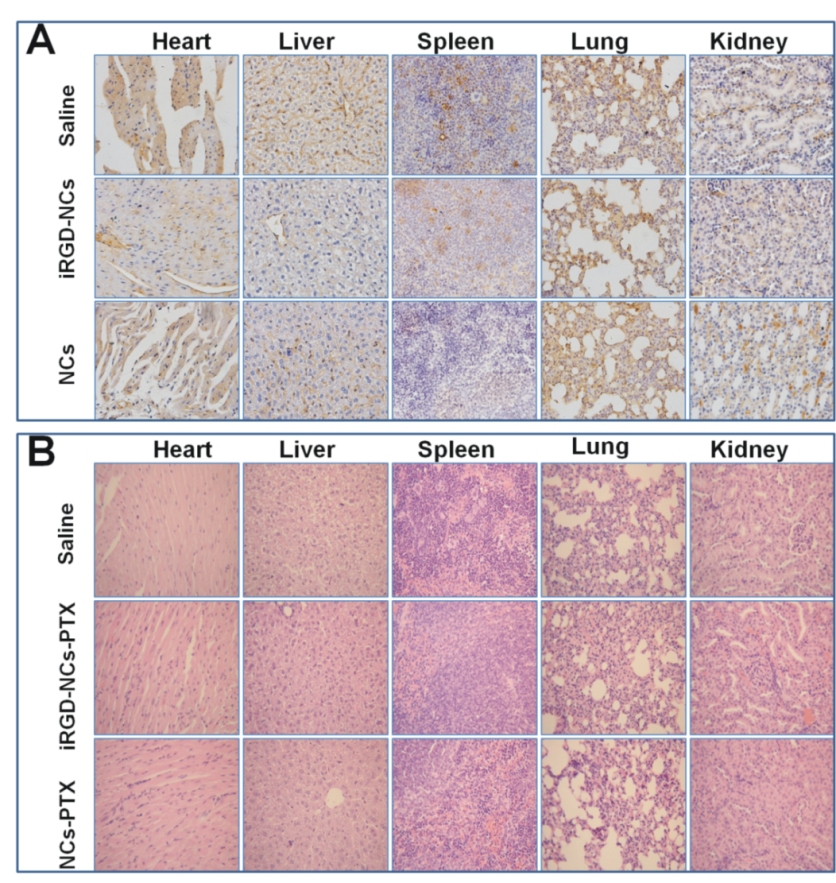


**Figure S8. (A)** CD68 immunohistological sections of healthy tissues (heart, liver, spleen, lung and kidney). The mice were injected with blank iRGD-NCs and NCs at the time points of 1, 4, 7 and 11 days, and saline was used as a control. The brown-stained area indicates the positive area. **(B)** H&E-stained tissue (heart, liver, spleen, lung and kidney) sections. The mice were treated with PTX-loaded NCs and iRGD-NCs at the time points of 1, 4, 7 and 11 days at a dose of 10 mg/kg PTX. The nucleus is bluish violet, and the endochylema is pink.
